# Crystal structure of 2-(4-fluoro-3-methyl­phen­yl)-5-{[(naphthalen-1-yl)­oxy]meth­yl}-1,3,4-oxa­diazole

**DOI:** 10.1107/S2056989015004144

**Published:** 2015-03-11

**Authors:** Muniyappan Govindhan, Kathavarayan Subramanian, Vijayan Viswanathan, Devadasan Velmurugan

**Affiliations:** a Department of Chemistry, Anna University, Chennai 600 025, India; b Orchid Chemicals & Pharmaceuticals Ltd, R&D Centre, Sholinganallur, Chennai 600 119, India; c Centre of Advanced Study in Crystallography and Biophysics, University of Madras, Guindy Campus, Chennai 600 025, India

**Keywords:** crystal structure, triazole, oxa­diazole, naphthalene, hydrogen bonding, π–π inter­actions

## Abstract

The title compound, C_20_H_15_FN_2_O_2_, adopts an almost planar conformation. The oxa­diazole ring makes dihedral angles of 13.90 (1) and 7.93 (1)° with the naphthalene ring system and benzene ring, respectively, while the naphthalene ring system and benzene ring are inclined to one another by 6.35 (1)°. In the crystal, adjacent mol­ecules are linked *via* C—H⋯N hydrogen bonds, forming chains propagating along [100]. There are also π–π inter­actions present [inter­centroid distances = 3.5754 (9) and 3.7191 (12) Å], linking the chains to form ribbons lying parallel to (011).

## Related literature   

For the biological activities of triazole derivatives, see: Desai *et al.* (2014 ▸), Khalilullah *et al.* (2012 ▸), Bethge *et al.* (2005 ▸); Saha *et al.* (2013 ▸); Shailaja *et al.* (2010 ▸); Sun *et al.* (2013 ▸).
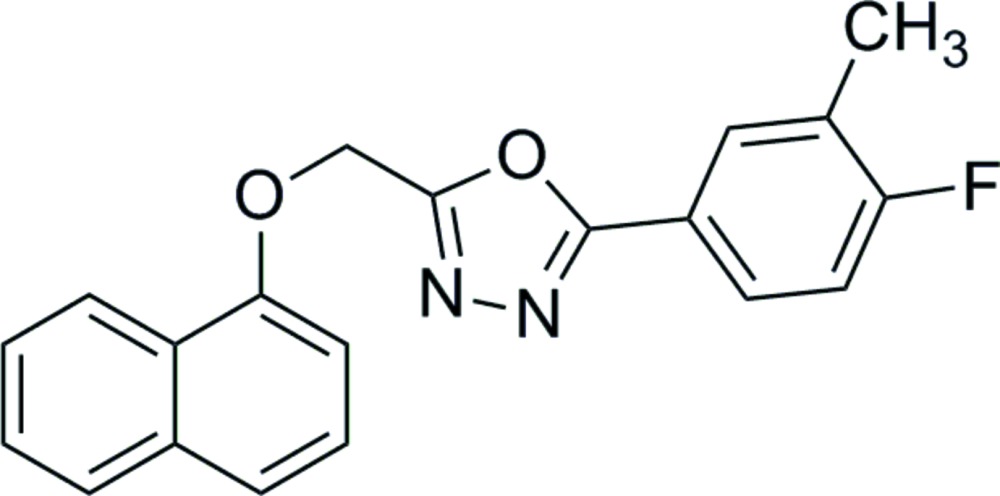


## Experimental   

### Crystal data  

C_20_H_15_FN_2_O_2_
*M* *_r_* = 334.34
Triclinic, 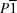

*a* = 7.4236 (4) Å
*b* = 7.5062 (4) Å
*c* = 16.3519 (8) Å
α = 77.092 (3)°
β = 77.494 (3)°
γ = 66.734 (3)°
*V* = 807.51 (7) Å^3^
*Z* = 2
Mo *K*α radiation
μ = 0.10 mm^−1^
*T* = 293 K
0.20 × 0.15 × 0.10 mm

### Data collection  

Bruker SMART APEXII area-detector diffractometer
Absorption correction: multi-scan (*SADABS*; Bruker, 2008 ▸) *T*_min_ = 0.981, *T*_max_ = 0.990
11476 measured reflections
3368 independent reflections
2306 reflections with *I* > 2σ(*I*)
*R*_int_ = 0.030

### Refinement  

*R*[*F* ^2^ > 2σ(*F* ^2^)] = 0.042
*wR*(*F* ^2^) = 0.134
*S* = 1.05
3368 reflections
227 parameters
H-atom parameters constrained
Δρ_max_ = 0.16 e Å^−3^
Δρ_min_ = −0.22 e Å^−3^

### 

Data collection: *APEX2* (Bruker, 2008 ▸); cell refinement: *SAINT* (Bruker, 2008 ▸); data reduction: *SAINT*; program(s) used to solve structure: *SHELXS97* (Sheldrick, 2008 ▸); program(s) used to refine structure: *SHELXL97* (Sheldrick, 2008 ▸); molecular graphics: *ORTEP-3 for Windows* (Farrugia, 2012 ▸); software used to prepare material for publication: *SHELXL97* and *PLATON* (Spek, 2009 ▸).

## Supplementary Material

Crystal structure: contains datablock(s) global, I. DOI: 10.1107/S2056989015004144/su5076sup1.cif

Structure factors: contains datablock(s) I. DOI: 10.1107/S2056989015004144/su5076Isup2.hkl

Supporting information file. DOI: 10.1107/S2056989015004144/su5076Isup3.cml

**Fig. 1.**
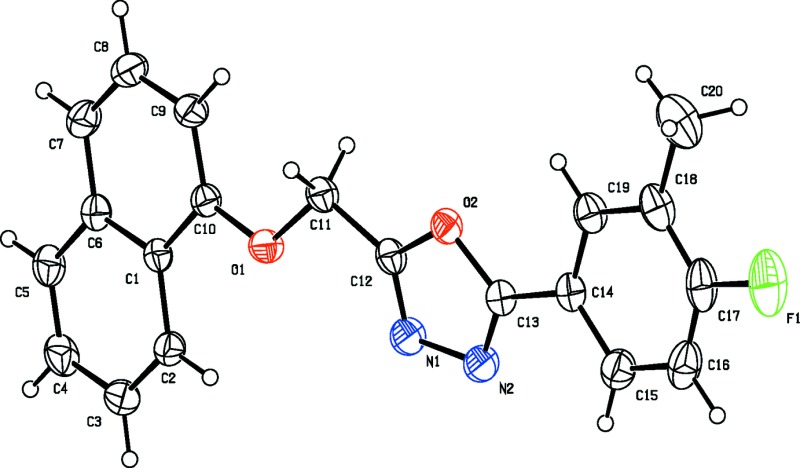
The molecular structure of the title compound, showing the atom labelling. Displacement ellipsoids are drawn at the 30% probability level.

**Fig. 2.**
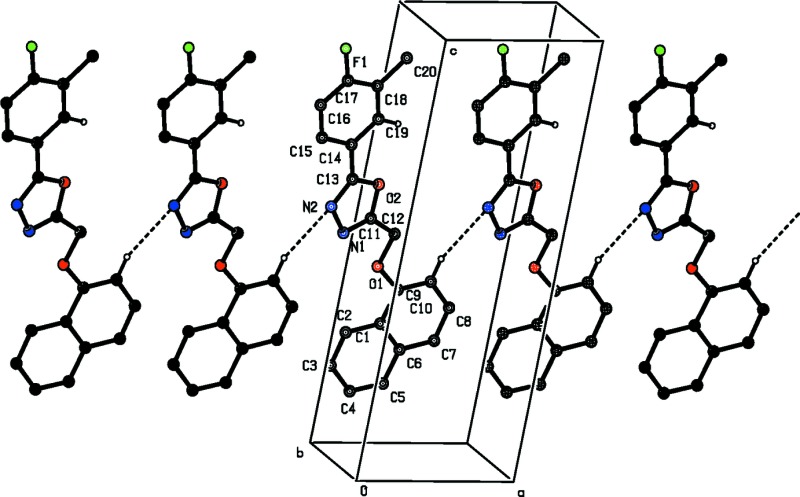
The crystal packing of the title compound, viewed along the b axis. Hydrogen bonds are shown as dashed lines (see Table 1 for details). H atoms not involved in hydrogen bonds have been omitted for clarity.

. DOI: 10.1107/S2056989015004144/su5076fig1.tif
The mol­ecular structure of the title compound, showing the atom labelling. Displacement ellipsoids are drawn at the 30% probability level.

b . DOI: 10.1107/S2056989015004144/su5076fig2.tif
The crystal packing of the title compound, viewed along the *b* axis. Hydrogen bonds are shown as dashed lines (see Table 1 for details). H atoms not involved in hydrogen bonds have been omitted for clarity.

CCDC reference: 1051477

Additional supporting information:  crystallographic information; 3D view; checkCIF report

## Figures and Tables

**Table 1:** Hydrogen-bond geometry (, )

*D*H*A*	*D*H	H*A*	*D* *A*	*D*H*A*
C9H9N2^i^	0.93	2.60	3.447(2)	152

Symmetry code: (i) 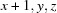.

## supplementary crystallographic information

### S1. Comment

1,3,4-Oxadiazole derivatives as typical heterocyclic compounds, exhibit a broad spectrum of biological activities and have polymer and material sciences application (Shailaja *et al.*, 2010) and are vital leading compounds for the development of drugs (Sun *et al.*, 2013). The presence of the oxadiazole motif in diverse types of compounds proves its importance in the field of medicinal chemistry (Saha *et al.*, 2013).

1,3,4-oxadiazole including anti-inflammatory, analgesic, anti-HIV, antimycobacterial, cathepsin K inhibitors, tyrosinase inhibitors, monoamine oxidase (MAO) inhibitors (Desai *et al.*, 2014), anticonvulsant, anticancer, antifungal, tuberculostatic (Khalilullah *et al.*, 2012), analgesic, antiplatelet and antithrombotic activities (Bethge *et al.*, 2005). Moreover the amino compounds are very commonly used as antimicrobial and germicidal drug.

The molecular structure of the title compound is shown in Fig. 1. The oxadiazole, naphthalene and flurophenyl rings adopt an almost planer conformation. The oxadiazole ring (C12/N1/N2/C13/O2) makes dihedral angles of 13.90 (1) ° and 7.93 (1) ° with naphthalene (C1-C10) and benzene ring (C14-C19/F1). The naphthalene ring makes a dihedral angle of 6.35 (1) ° with the benzene ring. The fluorine atom F1 and the methyl group C20 atom lie in the plane of the benzene ring to which they are attached [deviations from the benzene ring plane are 0.004 (1) and 0.003 (3) Å, respectively].

In the crystal, adjacent molecules are linked via C-H···N hydrogen bonds forming chains propagating along [100]; see Table 1 and Fig. 2. The chains are linked by π-π interactions, forming ribbons lying parallel to (011) [Cg1··· Cg1^i^ = 3.5754 (9) Å; inter-planar distance = 3.2669 (6) Å, slippage = 1.453 Å; Cg2···Cg4^i^ = 3.7191 (12) Å; Cg1, Cg2 and Cg4 are the centroids of rings O2/N1/N2/C12/C13, C1-C6 and C14-C19, respectively; symmetry code: (i) -x, -y+1, -z+1].

### S2. Experimental

Iodobenzene diacetate (2.0 mol eq) was added to a solution of naphthalen -1-yloxy-acetic acid (4-fluoro-3-methyl-benzylidene)-hydrazide (1.0 mole eq) in dioxane (10mL) at 298 - 303 K, and stirred at the same temperature for 15-30 min. Completion of the reaction was confirmed by TLC (Mobile phase Ethyl acetate/hexane, 3:7) and dioxane was distilled off in a rota-vapor. The obtained residue was dissolved in ethyl acetate and washed with saturated sodium bicarbonate solution, followed by water and brine solution. The organic layer was collected and dried over anhydrous sodium sulphate and distilled under vacuum. The crude product obtained was purified by column chromatography over silica gel (60-120 mesh) using hexane and ethyl acetate as a eluent to afford the title compound as an off-white solid. It was crystallised in methanol by slow evaporation giving colourless block-like crystals.

### S3. Refinement

The H atoms were placed in calculated positions and refined as riding atoms: C—H = 0.93 - 0.97 Å, with *U*_iso_(H) = 1.5*U*_eq_(C) for methyl H atoms and = 1.2*U*_eq_(C) for other H atoms.

### Crystal data

**Table:**

C_20_H_15_FN_2_O_2_	*Z* = 2
*M**_r_* = 334.34	*F*(000) = 348
Triclinic, *P*1	*D*_x_ = 1.375 Mg m^−^^3^
Hall symbol: -P 1	Mo *K*α radiation, λ = 0.71073 Å
*a* = 7.4236 (4) Å	Cell parameters from 3368 reflections
*b* = 7.5062 (4) Å	θ = 1.3–26.6°
*c* = 16.3519 (8) Å	µ = 0.10 mm^−^^1^
α = 77.092 (3)°	*T* = 293 K
β = 77.494 (3)°	Block, colourless
γ = 66.734 (3)°	0.20 × 0.15 × 0.10 mm
*V* = 807.51 (7) Å^3^	

### Data collection

**Table:**

Bruker SMART APEXII area-detector diffractometer	3368 independent reflections
Radiation source: fine-focus sealed tube	2306 reflections with *I* > 2σ(*I*)
Graphite monochromator	*R*_int_ = 0.030
ω and φ scans	θ_max_ = 26.6°, θ_min_ = 1.3°
Absorption correction: multi-scan (*SADABS*; Bruker, 2008)	*h* = −9→9
*T*_min_ = 0.981, *T*_max_ = 0.990	*k* = −9→9
11476 measured reflections	*l* = −20→20

### Refinement

**Table:**

Refinement on *F*^2^	Primary atom site location: structure-invariant direct methods
Least-squares matrix: full	Secondary atom site location: difference Fourier map
*R*[*F*^2^ > 2σ(*F*^2^)] = 0.042	Hydrogen site location: inferred from neighbouring sites
*wR*(*F*^2^) = 0.134	H-atom parameters constrained
*S* = 1.05	*w* = 1/[σ^2^(*F*_o_^2^) + (0.0629*P*)^2^ + 0.1875*P*] where *P* = (*F*_o_^2^ + 2*F*_c_^2^)/3
3368 reflections	(Δ/σ)_max_ = 0.001
227 parameters	Δρ_max_ = 0.16 e Å^−^^3^
0 restraints	Δρ_min_ = −0.22 e Å^−^^3^

### Special details

**Table:**

Geometry. All esds (except the esd in the dihedral angle between two l.s. planes) are estimated using the full covariance matrix. The cell esds are taken into account individually in the estimation of esds in distances, angles and torsion angles; correlations between esds in cell parameters are only used when they are defined by crystal symmetry. An approximate (isotropic) treatment of cell esds is used for estimating esds involving l.s. planes.
Refinement. Refinement of F^2^ against ALL reflections. The weighted R-factor wR and goodness of fit S are based on F^2^, conventional R-factors R are based on F, with F set to zero for negative F^2^. The threshold expression of F^2^ > 2sigma(F^2^) is used only for calculating R-factors(gt) etc. and is not relevant to the choice of reflections for refinement. R-factors based on F^2^ are statistically about twice as large as those based on F, and R- factors based on ALL data will be even larger.

### Fractional atomic coordinates and isotropic or equivalent isotropic displacement parameters (Å^2^)

**Table:**

	*x*	*y*	*z*	*U*_iso_*/*U*_eq_	
C1	0.2815 (2)	0.9674 (2)	0.27299 (10)	0.0380 (4)	
C2	0.0947 (3)	1.0312 (3)	0.24700 (11)	0.0458 (4)	
H2	−0.0126	1.0179	0.2860	0.055*	
C3	0.0698 (3)	1.1126 (3)	0.16502 (12)	0.0559 (5)	
H3	−0.0549	1.1560	0.1486	0.067*	
C4	0.2302 (3)	1.1315 (3)	0.10517 (12)	0.0585 (5)	
H4	0.2119	1.1853	0.0492	0.070*	
C5	0.4113 (3)	1.0721 (3)	0.12833 (11)	0.0542 (5)	
H5	0.5162	1.0865	0.0881	0.065*	
C6	0.4443 (3)	0.9883 (3)	0.21265 (11)	0.0443 (4)	
C7	0.6314 (3)	0.9262 (3)	0.23862 (12)	0.0550 (5)	
H7	0.7384	0.9387	0.1993	0.066*	
C8	0.6573 (3)	0.8484 (3)	0.32022 (13)	0.0572 (5)	
H8	0.7817	0.8093	0.3363	0.069*	
C9	0.4991 (3)	0.8257 (3)	0.38106 (11)	0.0486 (4)	
H9	0.5184	0.7738	0.4371	0.058*	
C10	0.3179 (2)	0.8801 (2)	0.35759 (10)	0.0400 (4)	
C11	0.1845 (3)	0.7566 (3)	0.49454 (10)	0.0460 (4)	
H11A	0.2094	0.8342	0.5279	0.055*	
H11B	0.2968	0.6338	0.4921	0.055*	
C12	0.0001 (3)	0.7192 (2)	0.53259 (10)	0.0427 (4)	
C13	−0.1993 (3)	0.6253 (2)	0.62939 (11)	0.0430 (4)	
C14	−0.2765 (3)	0.5508 (2)	0.71406 (11)	0.0444 (4)	
C15	−0.4706 (3)	0.5567 (3)	0.72898 (13)	0.0577 (5)	
H15	−0.5487	0.6074	0.6857	0.069*	
C16	−0.5456 (3)	0.4863 (3)	0.80897 (15)	0.0678 (6)	
H16	−0.6747	0.4885	0.8203	0.081*	
C17	−0.4279 (4)	0.4138 (3)	0.87090 (13)	0.0653 (6)	
C18	−0.2365 (4)	0.4046 (3)	0.85990 (12)	0.0590 (5)	
C19	−0.1633 (3)	0.4763 (3)	0.77889 (11)	0.0505 (5)	
H19	−0.0341	0.4736	0.7683	0.061*	
C20	−0.1144 (4)	0.3226 (4)	0.93077 (13)	0.0871 (8)	
H20A	−0.1727	0.4033	0.9747	0.131*	
H20B	0.0176	0.3204	0.9098	0.131*	
H20C	−0.1096	0.1915	0.9533	0.131*	
F1	−0.5067 (3)	0.3450 (2)	0.94919 (8)	0.0999 (6)	
N1	−0.1472 (2)	0.7432 (2)	0.49750 (9)	0.0555 (4)	
N2	−0.2797 (2)	0.6806 (2)	0.56191 (9)	0.0545 (4)	
O1	0.15466 (17)	0.86032 (18)	0.41174 (7)	0.0494 (3)	
O2	−0.01882 (18)	0.64483 (17)	0.61616 (7)	0.0440 (3)	

### Atomic displacement parameters (Å^2^)

**Table:**

	*U*^11^	*U*^22^	*U*^33^	*U*^12^	*U*^13^	*U*^23^
C1	0.0433 (10)	0.0364 (8)	0.0348 (8)	−0.0165 (7)	−0.0051 (7)	−0.0032 (6)
C2	0.0467 (11)	0.0496 (10)	0.0438 (10)	−0.0229 (9)	−0.0085 (8)	−0.0004 (8)
C3	0.0602 (13)	0.0593 (12)	0.0522 (11)	−0.0256 (10)	−0.0218 (9)	0.0038 (9)
C4	0.0791 (15)	0.0614 (12)	0.0368 (10)	−0.0317 (11)	−0.0148 (9)	0.0066 (8)
C5	0.0635 (13)	0.0584 (12)	0.0397 (10)	−0.0297 (10)	−0.0001 (9)	0.0012 (8)
C6	0.0482 (11)	0.0431 (9)	0.0416 (9)	−0.0203 (8)	−0.0020 (8)	−0.0040 (7)
C7	0.0457 (11)	0.0655 (12)	0.0529 (11)	−0.0267 (10)	0.0010 (8)	−0.0028 (9)
C8	0.0449 (11)	0.0699 (13)	0.0590 (12)	−0.0262 (10)	−0.0115 (9)	−0.0007 (10)
C9	0.0493 (11)	0.0568 (11)	0.0413 (9)	−0.0225 (9)	−0.0141 (8)	0.0024 (8)
C10	0.0416 (10)	0.0428 (9)	0.0353 (8)	−0.0175 (8)	−0.0043 (7)	−0.0026 (7)
C11	0.0509 (11)	0.0511 (10)	0.0330 (9)	−0.0198 (9)	−0.0069 (7)	0.0028 (7)
C12	0.0540 (11)	0.0421 (9)	0.0311 (8)	−0.0189 (8)	−0.0070 (7)	−0.0003 (7)
C13	0.0476 (11)	0.0390 (9)	0.0421 (10)	−0.0176 (8)	−0.0045 (8)	−0.0043 (7)
C14	0.0532 (11)	0.0383 (9)	0.0402 (9)	−0.0188 (8)	0.0002 (8)	−0.0055 (7)
C15	0.0552 (12)	0.0541 (11)	0.0633 (13)	−0.0235 (10)	−0.0006 (9)	−0.0088 (9)
C16	0.0651 (14)	0.0620 (13)	0.0748 (15)	−0.0338 (12)	0.0202 (12)	−0.0167 (11)
C17	0.0882 (17)	0.0523 (12)	0.0489 (12)	−0.0346 (12)	0.0231 (11)	−0.0099 (9)
C18	0.0824 (16)	0.0473 (11)	0.0391 (10)	−0.0205 (11)	0.0018 (9)	−0.0066 (8)
C19	0.0581 (12)	0.0486 (10)	0.0405 (10)	−0.0197 (9)	0.0009 (8)	−0.0057 (8)
C20	0.123 (2)	0.0851 (17)	0.0403 (12)	−0.0314 (16)	−0.0105 (13)	0.0024 (11)
F1	0.1377 (14)	0.0909 (10)	0.0602 (8)	−0.0597 (10)	0.0404 (8)	−0.0094 (7)
N1	0.0603 (11)	0.0690 (11)	0.0386 (8)	−0.0302 (9)	−0.0109 (7)	0.0055 (7)
N2	0.0563 (10)	0.0671 (10)	0.0434 (9)	−0.0298 (9)	−0.0114 (7)	0.0031 (7)
O1	0.0425 (7)	0.0639 (8)	0.0348 (6)	−0.0199 (6)	−0.0047 (5)	0.0066 (5)
O2	0.0510 (8)	0.0498 (7)	0.0314 (6)	−0.0222 (6)	−0.0061 (5)	0.0010 (5)

### Geometric parameters (Å, º)

**Table:**

C1—C2	1.405 (2)	C11—H11B	0.9700
C1—C6	1.422 (2)	C12—N1	1.276 (2)
C1—C10	1.426 (2)	C12—O2	1.3552 (19)
C2—C3	1.363 (2)	C13—N2	1.277 (2)
C2—H2	0.9300	C13—O2	1.371 (2)
C3—C4	1.401 (3)	C13—C14	1.456 (2)
C3—H3	0.9300	C14—C19	1.376 (3)
C4—C5	1.349 (3)	C14—C15	1.393 (3)
C4—H4	0.9300	C15—C16	1.381 (3)
C5—C6	1.412 (2)	C15—H15	0.9300
C5—H5	0.9300	C16—C17	1.359 (3)
C6—C7	1.408 (3)	C16—H16	0.9300
C7—C8	1.354 (3)	C17—F1	1.367 (2)
C7—H7	0.9300	C17—C18	1.368 (3)
C8—C9	1.405 (3)	C18—C19	1.393 (3)
C8—H8	0.9300	C18—C20	1.497 (3)
C9—C10	1.357 (2)	C19—H19	0.9300
C9—H9	0.9300	C20—H20A	0.9600
C10—O1	1.376 (2)	C20—H20B	0.9600
C11—O1	1.418 (2)	C20—H20C	0.9600
C11—C12	1.482 (2)	N1—N2	1.412 (2)
C11—H11A	0.9700		
			
C2—C1—C6	119.18 (15)	H11A—C11—H11B	108.7
C2—C1—C10	123.22 (15)	N1—C12—O2	113.17 (16)
C6—C1—C10	117.60 (16)	N1—C12—C11	129.27 (15)
C3—C2—C1	120.36 (17)	O2—C12—C11	117.55 (15)
C3—C2—H2	119.8	N2—C13—O2	112.28 (15)
C1—C2—H2	119.8	N2—C13—C14	128.39 (17)
C2—C3—C4	120.63 (19)	O2—C13—C14	119.32 (15)
C2—C3—H3	119.7	C19—C14—C15	119.73 (17)
C4—C3—H3	119.7	C19—C14—C13	121.76 (17)
C5—C4—C3	120.33 (17)	C15—C14—C13	118.51 (18)
C5—C4—H4	119.8	C16—C15—C14	119.1 (2)
C3—C4—H4	119.8	C16—C15—H15	120.4
C4—C5—C6	121.17 (17)	C14—C15—H15	120.4
C4—C5—H5	119.4	C17—C16—C15	119.0 (2)
C6—C5—H5	119.4	C17—C16—H16	120.5
C7—C6—C5	122.32 (17)	C15—C16—H16	120.5
C7—C6—C1	119.36 (16)	C16—C17—F1	117.4 (2)
C5—C6—C1	118.31 (17)	C16—C17—C18	124.44 (19)
C8—C7—C6	120.77 (17)	F1—C17—C18	118.2 (2)
C8—C7—H7	119.6	C17—C18—C19	115.8 (2)
C6—C7—H7	119.6	C17—C18—C20	121.8 (2)
C7—C8—C9	121.02 (18)	C19—C18—C20	122.3 (2)
C7—C8—H8	119.5	C14—C19—C18	121.9 (2)
C9—C8—H8	119.5	C14—C19—H19	119.0
C10—C9—C8	119.62 (17)	C18—C19—H19	119.0
C10—C9—H9	120.2	C18—C20—H20A	109.5
C8—C9—H9	120.2	C18—C20—H20B	109.5
C9—C10—O1	124.18 (15)	H20A—C20—H20B	109.5
C9—C10—C1	121.59 (16)	C18—C20—H20C	109.5
O1—C10—C1	114.23 (14)	H20A—C20—H20C	109.5
O1—C11—C12	106.29 (14)	H20B—C20—H20C	109.5
O1—C11—H11A	110.5	C12—N1—N2	105.94 (14)
C12—C11—H11A	110.5	C13—N2—N1	106.37 (15)
O1—C11—H11B	110.5	C10—O1—C11	117.26 (13)
C12—C11—H11B	110.5	C12—O2—C13	102.25 (13)
			
C6—C1—C2—C3	−0.1 (2)	O2—C13—C14—C15	171.45 (15)
C10—C1—C2—C3	−179.64 (16)	C19—C14—C15—C16	−0.3 (3)
C1—C2—C3—C4	0.8 (3)	C13—C14—C15—C16	179.90 (16)
C2—C3—C4—C5	−1.1 (3)	C14—C15—C16—C17	0.2 (3)
C3—C4—C5—C6	0.6 (3)	C15—C16—C17—F1	−179.84 (17)
C4—C5—C6—C7	−179.61 (19)	C15—C16—C17—C18	−0.1 (3)
C4—C5—C6—C1	0.2 (3)	C16—C17—C18—C19	0.2 (3)
C2—C1—C6—C7	179.40 (16)	F1—C17—C18—C19	179.88 (16)
C10—C1—C6—C7	−1.1 (2)	C16—C17—C18—C20	−179.9 (2)
C2—C1—C6—C5	−0.4 (2)	F1—C17—C18—C20	−0.2 (3)
C10—C1—C6—C5	179.18 (15)	C15—C14—C19—C18	0.3 (3)
C5—C6—C7—C8	179.28 (18)	C13—C14—C19—C18	−179.86 (16)
C1—C6—C7—C8	−0.5 (3)	C17—C18—C19—C14	−0.3 (3)
C6—C7—C8—C9	0.6 (3)	C20—C18—C19—C14	179.80 (18)
C7—C8—C9—C10	0.9 (3)	O2—C12—N1—N2	0.0 (2)
C8—C9—C10—O1	178.61 (16)	C11—C12—N1—N2	178.53 (17)
C8—C9—C10—C1	−2.5 (3)	O2—C13—N2—N1	−0.2 (2)
C2—C1—C10—C9	−177.89 (17)	C14—C13—N2—N1	178.90 (16)
C6—C1—C10—C9	2.6 (2)	C12—N1—N2—C13	0.1 (2)
C2—C1—C10—O1	1.1 (2)	C9—C10—O1—C11	−7.4 (2)
C6—C1—C10—O1	−178.44 (14)	C1—C10—O1—C11	173.68 (14)
O1—C11—C12—N1	10.3 (3)	C12—C11—O1—C10	−168.05 (14)
O1—C11—C12—O2	−171.25 (13)	N1—C12—O2—C13	−0.12 (19)
N2—C13—C14—C19	172.65 (18)	C11—C12—O2—C13	−178.81 (14)
O2—C13—C14—C19	−8.3 (2)	N2—C13—O2—C12	0.17 (18)
N2—C13—C14—C15	−7.5 (3)	C14—C13—O2—C12	−178.98 (14)

### Hydrogen-bond geometry (Å, º)

**Table:**

*D*—H···*A*	*D*—H	H···*A*	*D*···*A*	*D*—H···*A*
C9—H9···N2^i^	0.93	2.60	3.447 (2)	152

Symmetry code: (i) *x*+1, *y*, *z*.
