# Supplementary figures and images for: Crystal structure of 2-(4-fluoro-3-methyl­phen­yl)-5-{[(naphthalen-1-yl)­oxy]meth­yl}-1,3,4-oxa­diazole
Source: Acta Crystallogr E Crystallogr Commun. 2015 Mar 11;71(Pt 4):o229–30. doi: 10.1107/S2056989015004144 (PMC4438827; doi:10.1107/S2056989015004144)

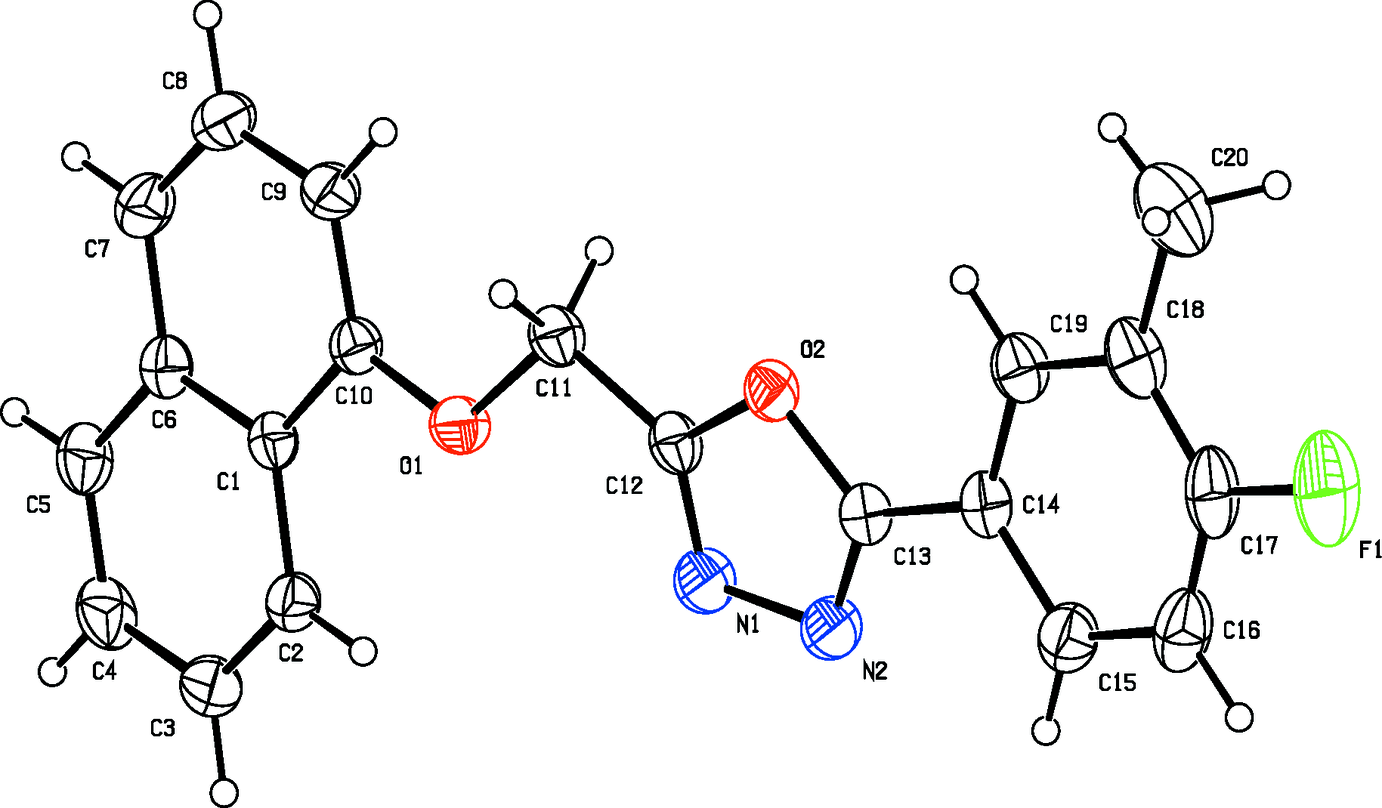

Supplement: Supplementary file 4 [file e-71-0o229-fig1.tif]

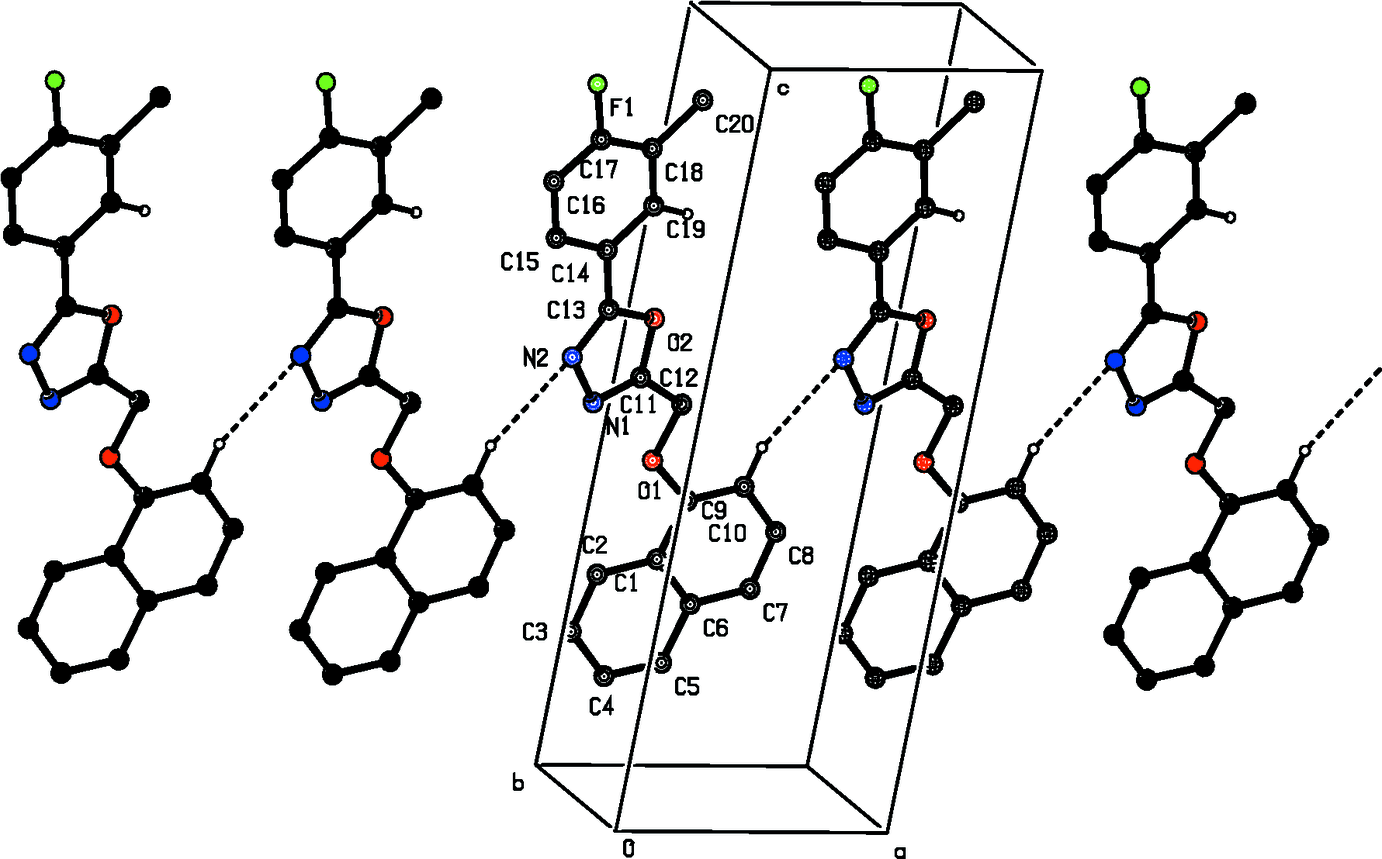

Supplement: Supplementary file 5 [file e-71-0o229-fig2.tif]
